# Factors underlying burnout among rural village physicians in Southwestern China

**DOI:** 10.1002/hcs2.62

**Published:** 2023-07-26

**Authors:** Xingyue Zhu, Yang Chen, Xingjiang Liao

**Affiliations:** ^1^ School of Medicine and Health Management Guizhou Medical University Guiyang Guizhou China; ^2^ Center of Medicine Economics and Management Research Guizhou Medical University Guiyang Guizhou China; ^3^ Department of Hospital Quality Evaluation and Medical Record Management The Third People's Hospital of Chengdu Chengdu Sichuan China

**Keywords:** village physician, medical alliance, burnout, Oldenburg Burnout Inventory, rural area

## Abstract

**Background:**

Primary healthcare doctors in China often experience problems with occupational burnout, a condition known to relate to high job stress and low wages. In China, many medical alliances have recently been established in rural areas, where village physicians work as healthcare gatekeepers. However, burnout in village physicians in the context of medical alliances remains underresearched.

**Methods:**

This cross‐sectional survey was conducted among 100 village physicians practicing at village clinics in Qiandongnan prefecture, Guizhou province, China. An online questionnaire was distributed to assess physicians' demographic characteristics and work situations. Burnout was measured using the Oldenburg Burnout Inventory (validated Chinese version). A multivariate linear model with stepwise procedure was used to estimate the effects of factors of interest on burnout, focusing particularly on actions within the medical alliance that involved respondents' clinics, such as training and support for village physicians provided by higher‐level facilities.

**Results:**

The overall response rate was 79%. The mean burnout score was 38.09 (standard deviation, 4.55; range, 25–47). The multivariate analysis showed that fewer working years and too much farming work were significantly related to exacerbation of burnout. Greater medical services in the total workload and greater support from higher‐level facilities were associated with burnout alleviation.

**Conclusion:**

Close connections and interactions across medical alliance member facilities could facilitate reduction in burnout for village physicians practicing as primary care gatekeepers.

## Graphical abstract

This study investigated the factors on burnout of village physicians in China, particularly exploring the effects of the medical alliance. Fewer working years, too much farming work and less workload of medical services were significantly related to exacerbation of burnout. While more supports from higher‐level facilities, including transmission of images and testing results across facilities and tele‐consultations, were conducive to burnout alleviation.

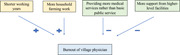

**Abbreviations**
AIC: Akaike Information Criterion
*CI*
: confidence interval
GDP: gross domestic product
OLBI: Oldenburg Burnout Inventory
Q‐Q plot: quantile‐quantile plot
*SD*
: standard deviation

## 1 BACKGROUND

Fragmented delivery of healthcare and insufficient capacity of primary facilities are major contributors to inefficiencies in the healthcare system in China [1]. In 2015, China initiated efforts to integrate the healthcare delivery system. In this system, primary healthcare facilities provide primary general medical care and prevention, and secondary and tertiary hospitals provide specialty medicine care; services across facilities at different levels are coordinated [2]. The rationale underlying the tiered health system is the division of work, as primary care and specialty care are two products with distinct characteristics and different scopes of application. Therefore, the tiered health system makes it possible to provide healthcare while saving expenditure by providing access to services that are appropriate to different needs. The healthcare delivery system in China has traditionally been hospital‐centric and treatment‐based [3, 4]. A complete and effective tiered health system relies more on adequate staffing by competent generalists at the primary level and the commensurate infrastructure. However, understaffing and poor infrastructure are persistent issues in primary care facilities, particularly in rural areas with weaker economies [5, 6].

Guizhou province, located in the southwest of China, ranked 28th out of 31 provinces on GDP per capita in 2021; medical resources in the province are very scarce and poorly distributed [7]. To provide essential access to healthcare and medical services using the limited resources in Guizhou, reforms in the healthcare delivery system are vital. Therefore, Guizhou has been exploring the possibility of close‐knit medical alliances within county jurisdiction since May 2019, to integrate the medical resources in the county, increase the capacity of primary care providers, and deliver accessible, reasonable‐quality care in a more efficient fashion. An ideal close‐knit medical alliance is a consortium with aligned interests that has a unified management across its member facilities, which requires the reorganization of the management authority. This structure is likely to improve efficiency and access. As of July 2022, Guizhou province had established close‐knit medical alliances in 81 counties. These medical alliances are led by the county hospital (the top tier in the alliance), and include rural township health centers (the middle tier) and village clinics (the bottom tier) within the county jurisdiction. The management of personnel, financial affairs, performance evaluation, and other key elements within the alliance is shared. The integration of medical resources within a county may be more feasible for rural areas with lower population density; this close‐knit organization is designed to facilitate consistent incentives and increase efficiency. In such medical alliances, rural physicians in village clinics act as healthcare gatekeepers and are therefore accountable for addressing the primary healthcare needs of individuals. Ideally, primary care physicians provide the following gatekeeping services: first contact access, long‐term care for chronic diseases, comprehensive services for common conditions, and coordinated services across levels [8]. Accordingly, village physicians undertake vital tasks, and understanding how they feel about their occupation is important in promoting better performance.

Burnout is a common issue among physicians and is not only detrimental to physicians' well‐being but a threat to patient health [9, 10]. High burnout in doctors has hindered systematic reform in the Chinese health sector [11]. Severe burnout experienced by Chinese rural practitioners in township health centers relates to low job satisfaction, long working hours, and performance‐based salary [12, 13]. However, the effect of additional stress‐related and mitigating factors on burnout in physicians in rural village clinics in China are yet to be investigated, particularly in the context of the current tiered health system. In this study, we attempted to delineate the workload and identify predictors of burnout for physicians at village clinics in rural regions. A questionnaire survey was conducted in the southwestern province of Guizhou, China. We hope that the findings help to provide a foundation for further improvement in the existing tiered health system.

## 2 METHODS

### 2.1 Participants

Our survey was based on the 95th China village physician training session sponsored by the Red Cross Society of China. This training session was open only to village physicians in Qiandongnan prefecture, located in southeast Guizhou province, and it was held by one medical college from August 26, 2022, to September 8, 2022. Each township health center in Qiandongnan prefecture recommended for the training one to two excellent village physicians who had not previously received village physician training sessions by the Red Cross Society of China. The participants were ultimately selected by the Red Cross Society of Guizhou. In total, 100 village physicians from 100 villages in the 16 counties in Qiandongnan prefecture took part in the training, and were then invited to participate in our survey.

### 2.2 Procedure

An electronic questionnaire was sent to all 100 village physicians via the social media app WeChat. The questionnaire comprised two sections. The first section assessed the following main individual characteristics and basic work characteristics: sex, education level, working years, professional titles; and workload, load composition, load for other productive work (farming), performance bonus, and training and support from higher‐level facilities. The second section measured burnout. The survey was conducted using the network platform Questionnaire Star (http://www.wjx.cn) on September 18, 2022.

We used the Oldenburg Burnout Inventory (OLBI) to measure burnout in the respondents. The OLBI is one of the most widely used tools to assess occupational burnout, which is defined by the two dimensions of exhaustion and disengagement [14, 15]. This inventory contains 16 items scored on a scale ranging from 1 to 4 (strongly agree to strongly disagree). The score on each item is summed to obtain a total burnout score. The original OLBI was in German, and recent research has evaluated the validity and reliability of the Chinese version of the OLBI [16]. We used the validated Chinese version to develop our questionnaire.

### 2.3 Statistical analysis

Descriptive statistics were used to characterize the survey results. Mean and standard deviation was used for numerical variables, and frequency and proportion was used for categorical variables. To examine the factors related to professional burnout in more depth, a multivariate linear regression model was constructed. The included covariates were demographic characteristics and work characteristics assessed in the first section of the questionnaire. Whether and how close the connections are between member facilities in the alliance is believed to affect the work of primary care providers; however, this factor cannot be measured using only a survey of village physicians. Therefore, we used training times and the number of support activities that village physicians received as proxies for organizational effects. We expected that there would be more interactions between the member facilities if they had closer connections. We also used the stepwise procedure to determine a subset of covariates to reduce the possibility of multicollinearity and overspecification. The Akaike information criterion was used to compare the full model and the stepwise model. To control for potential heterogeneity from different counties, clustered standard errors at county groups were estimated. In addition, we conducted visual inspection to ensure that our linear regression model satisfied the assumption of normally distributed residuals. The significance level was set at 0.05 for two‐sided tests. We used Stata 15 (StataCorp LP) to conduct the statistical analysis.

## 3 RESULTS

### 3.1 Close‐knit medical alliances

**Figure 1:**
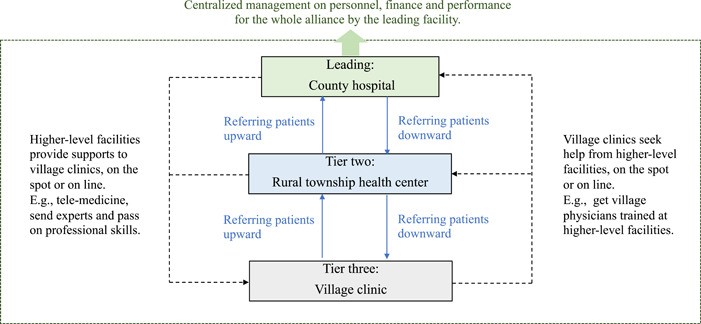
Structure of a close‐knit medical alliance.

All the counties in Qiandongnan prefecture have created close‐knit medical alliances. The operation of these alliances is shown in Figure 1. To determine whether the facilities involved in an alliance are closely connected, the Guizhou Health Commission has developed an evaluation plan with four dimensions: responsibility establishment, management, care delivery, and economic interests. Responsibility establishment requires that the lead facility of an alliance (typically the county hospital) acts as the primary decision maker, and is responsible for formulating the alliance management policies and contracting payers regarding specific payment methods. The local government conducts the evaluation of the medical alliance. Regarding management, the alliance is granted autonomy to govern its internal affairs; for example, those related to personnel (performance evaluation, income distribution, and title determination), finance (unified management of finance and budget), and drugs and devices (identical utilization lists and unified procurement). For care delivery, the provided services should be coordinated across the alliance and information ought to be shared. Finally, the revenue from medical services should be subjected to unified management and independent accounting within the alliance, while subsidies of the basic public health services are issued to practitioners according to individual performance evaluation results.

### 3.2 Task list for village physicians

The National Health Commission stipulates the content of basic public health services, which comprise 12 items, all of which primary facilities must provide. Among these items, nursing care for neonates, pregnant women, older people, and patients with stable chronic conditions are primary foci. A detailed summary of each area and corresponding requirements are shown in Supporting Information: Table S1. The basic public health services are funded by the government and free of charge to all citizens, to ensure the provision of equitable and accessible healthcare services. Provision of the basic public health services is the responsibility of village physicians at village clinics, and such physicians constitute the foundation of the health system in rural regions. In Qiandongnan, physician performance of the basic public health services is evaluated each quarter. In addition to the basic public health services, village physicians may also provide medical services such as diagnoses and treatment of common diseases through the use of chemical drugs, nonmedication interventions, and traditional Chinese medicine.

### 3.3 Survey results

The overall response rate was 79% (79 out of 100 village physicians completed the questionnaire). Characteristics of the respondents are summarized in Table 1. Among all the respondents, there were slightly fewer men than women (45.57% vs. 54.43%), and the average age was 38.70 years (standard deviation, 8.13). The distribution of respondents across different working experience groups was relatively balanced: 27.85% had up to 5 years of working experience; 20.25% had 6 to 10 years; 29.11% had 11 to 20 years; and 22.78% had over 20 years. Only 12.66% of respondents had a bachelor's degree, and nearly half (34 [43.04%]) had no professional title. It is not uncommon for village physicians to be involved in household farm work, and a few respondents (5 [6.33%]) had to undertake farm work equivalent to at least one‐third of their village physician workload. Most respondents (45 [56.96%]) worked 50–74 h per week, and for most, basic public health services accounted for more of the total workload than medical services (69 [87.34%]). The income of most respondents was supplemented by a performance bonus (49 [62.03%]). Within the medical alliance, 44.30% of the respondents had frequent chances for professional training in higher‐level facilities, and 72.16% received some support from higher‐level facilities, such as special consultations at village clinics by more skilled physicians at higher levels, information sharing of testing and imaging as well as telemedicine within the alliance. The evaluation of burnout showed that the mean score was 38.09 (standard deviation, 4.55; range 25–47).

**Table 1:** Characteristics of the respondents (*N* = 79).

Characteristics	*N* (%)
Sex	
Male	36 (45.57)
Female	43 (54.43)
Age, *mean* ± *SD*, (years)	38.70 ± 8.13
Working years, (years)	
≤5	22 (27.85)
6–10	16 (20.25)
11–20	23 (29.11)
>20	18 (22.78)
Education level	
Technical school	33 (41.77)
Junior college	36 (45.57)
Bachelor degree	10 (12.66)
Title	
Professor village physician	1 (1.27)
Associate professor village physician	0
Village physician in charge	2 (2.53)
Assistant village physician	23 (29.11)
Other	19 (24.05)
No title	34 (43.04)
Workload for farm work	
<one‐third of workload for village physician	29 (36.71)
≥one‐third of workload for village physician	5 (6.33)
No farm work	45 (56.96)
Work hour per week (hours)	
40–49	19 (24.05)
50–74	45 (56.96)
≥75	15 (18.99)
Workload composition	
Basic public health service dominated	69 (87.34)
Medical service dominated	10 (12.66)
Performance bonus	
No	23 (29.11)
Yes	49 (62.03)
Unclear	7 (8.86)
No. of received trainings per year [time (s)]	
<1	19 (24.05)
1–3	25 (31.65)
>3	35 (44.30)
No. of received supports per year^a^ [time (s)]	
<1	22 (27.85)
1–5	49 (62.03)
>5	8 (10.13)
Burnout, *mean* ± *SD*	38.09 ± 4.55

Abbreviation: *SD*, standard deviation.

a Support included special consultations at village clinics by physicians from higher‐level facilities, sharing information about testing and imaging within the alliance, and telemedicine.

### 3.4 Factors related to burnout

**Figure 2:**
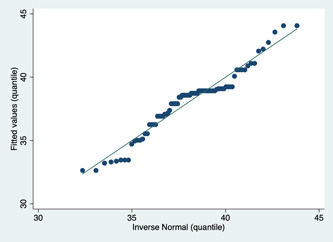
Q‐Q plot of residuals in the stepwise model.

The results of the stepwise regression on burnout score are shown in Table 2. Sex, educational level, professional title, and length of work hours were not associated with burnout score and were hence excluded from the model. Fewer work years was significantly related to higher burnout. Compared with minimal additional workload for household farm work, too much farm work increased perceived burnout for village physicians (coefficient = 4.28; 95% confidence interval, 0.49–8.07; *p* = 0.028). For workload structure, sharing more medical services was associated with burnout alleviation (coefficient = −3.71; 95% confidence interval, −6.36 to −1.06; *p* = 0.007). Performance bonus and training opportunities were excluded owing to their insignificant effects. However, greater support from higher‐level facilities significantly correlated with lower burnout score. The results of the full regression model are shown in Supporting Information: Table S2; in this model, the effects of working years, workload for farm work, workload composition, and the amount of received support per year remained significant. The Akaike information criterion suggested that the stepwise model explained a similar amount of variation but with much less information required. The residuals from the stepwise model are plotted in Figure 2, which indicates that the residuals were approximately normally distributed.

**Table 2:** Results of the stepwise linear regression on burnout score.

Characteristic	Coefficient	*p* Value	95% *CI*	
Working years (years)				
≤5	0[Reference]			
6–10	−2.67	0.041	−5.22	−0.12
11–20	−2.83	0.034	−5.44	−0.22
>20	−2.39	0.058	−4.86	0.08
Workload for farm work				
<one‐third of workload for village physician	0[Reference]			
≥one‐third of workload for village physician	4.28	0.028	0.49	8.07
No farm work	−1.66	0.093	−3.59	0.28
Workload composition				
Basic public health service dominated	0[Reference]			
Medical service dominated	−3.71	0.007	−6.36	−1.06
No. of received supports per year^a^ [time (s)]				
<1	0[Reference]			
1–5	−2.17	0.053	−4.37	0.03
>5	−5.79	0.016	−10.33	−1.24
$R^{2}$				0.3163
AIC	450.4195			

Abbreviations: AIC, Akaike information criterion; *CI*, confidence interval.

a Support included special consultations at village clinics by physicians from higher‐level facilities, sharing information about testing and imaging within the alliance, and telemedicine.

## 4 DISCUSSION

This study identified some important factors underlying burnout in village physicians in China. We found that more working years was associated with lower likelihood of burnout. Seniority can lead to the accumulation of work experience and familiarity with residents, which facilitates the work of village physicians. A substantial workload for other productive work was a stressor leading to burnout, as it diverted the energy of village physicians away from their professional duties. Village physicians constitute a disparate group of medical practitioners because many of them practice in their home town, and it is not uncommon for them to continue to help their families with farm work to some extent after they become physicians. The true workload status of village physicians, which includes in‐clinic duties and off‐duty work, requires more attention from medical alliance leaders. Village physicians in deprived areas tend to have a greater additional workload, which may suggest problems with underpayment and inadequate in‐service training.

Another interesting finding was that the proportion of medical services within the total workload had an effect on burnout. The provision of basic public health services, which have a prescribed scope and predetermined to‐do lists, can be associated with lower professional autonomy among primary healthcare providers [17]. Such autonomy provides a greater sense of personal accomplishment, thereby increasing enthusiasm for work, compared with step‐by‐step completion of lists and receiving quarterly performance evaluations. In addition, diagnostic and treatment services accord more with the public perception of physicians' responsibilities, perhaps because the provision of these medical services is assumed to constitute the work of physicians. For a village physician, the provision of basic public health services constitutes a fixed workload, whereas the provision of medical services is determined by many factors, including both physician aspects (ability to deal with common diseases) and patient aspects (willingness to contact the village physician when they require healthcare). Increasing village physicians' ability to provide medical services is very important to enable them to develop sustained partnership with patients at their clinics.

Although village physicians play a vital role in the tiered health system, how that system affects the health providers involved in terms of their professional experience remains to be determined. This study yielded preliminary evidence that support from higher‐level facilities to village clinics is associated with a lower level of burnout. The leading group in a close‐knit medical alliance usually experiences the pressure of evaluation performance of the whole alliance, which motivates the group to try to manage more patients in primary settings. Accordingly, it is very important for the alliance to increase the ability of practitioners in primary facilities, including village physicians at village clinics, to help them be more competent gatekeepers and provide nursing care and continuing follow‐up for vulnerable populations. Technical support and professional training are common tools to achieve this. However, we did not observe any positive effects from the training that village physicians underwent. Theoretically, support and training are both good ways to improve village physicians' professional skills and thus boost their work efficiency. However, training takes time and so might exacerbate physicians' exhaustion after work; additionally, there may be a lack of effective training for primary health workers [18, 19]. In contrast, support activities such as special consultations, telemedicine, and information sharing do not require much additional effort from village physicians. This may partly explain our results.

To further improve medical alliances, it is important to focus on the medical capacity of primary facilities while ensuring that the provision of basic public health services is adequate. The basic public health service list is a streamlined framework to help establish the role of gatekeeper for village physicians, but it does not address all the implications of the gatekeeping role. The promotion of physicians' ability to provide medical services benefits both village physicians (in terms of their work satisfaction) and the entire tiered health system (in terms of overall efficacy). However, provision of the basic public health services entails an enormous effort, and the increased requirements for medical capacity clearly add to the burden of village physicians. The issues of understaffing and lack of resources for managing patients in primary facilities, particularly in rural regions, require further research, and attention from policymakers.

Current evaluations of close‐knit medical alliances focus on the efficiency of care delivery, namely, the rate of patients referred downward and the rate of patients consulting at primary facilities. It is noteworthy that an effective medical alliance is based on adequate facilities at each tier. In the context of the relatively weak professional capacities of primary facilities, the development of primary care needs more attention. It is the responsibility of the alliance leadership and the local government to assist and nurture village physicians. Training and support for village physicians, as well as the ways in which these are implemented, are aspects of the close‐knit medical alliance that should be evaluated. More than 20% of the respondents received little training or support from higher‐level facilities. This may be attributed to insufficient awareness among the leadership of medical alliances, as evaluations of the alliance do not include requirements to increase the ability of primary care physicians.

Caution is needed when extrapolating these findings to other regions. Guizhou has a severe scarcity of medical resources, and primary care physicians in the province may be more likely to be undertrained. In this survey, we found that only 12.66% of the participating village physicians had a bachelor's degree. It is possible that our respondents were more sensitive to support activities. The results also indicate the need to develop tailored strategies to build and refine medical alliances in underdeveloped areas where primary facilities are characterized by insufficient workforce, poor ability, and high cost constraints. Online training courses can help if there is a dearth of local experts. To recruit students from rural regions as directed generalist candidates in medical colleges is another promising option. Research findings on the association between financial incentives and physicians' behaviors are ambiguous [20, 21]; similarly, we did not observe an effect of performance bonus on perceived burnout in village physicians. However, appropriate compensation can be helpful in attracting and retaining generalists in primary care settings. More strategies to develop payment models are needed.

This study had some limitations. The sample size was too small to permit a comprehensive estimate of the prevalence of burnout among village physicians, or to represent the situation of all village physicians in Guizhou. The small sample also precluded the investigation of additional factors related to burnout, for instance, the social security that village physicians receive and other characteristics associated with the operation of close‐knit medical alliances. Moreover, we did not consider training or support activities outside the medical alliance, such as those provided by continuing medical education programs and charity societies, which may have introduced confounding. Additional studies are needed to provide an overview of burnout in village physicians in China. The cross‐sectional data did not capture the dynamics of burnout over a period of time. Finally, the survey did not differentiate training and support in terms of their content. The type and content of these activities may have different effects on burnout.

## 5 CONCLUSIONS

Close connections and interactions across medical alliance member facilities may help to reduce burnout in village physicians, possibly by refining their professional. Further improvement in close‐knit medical alliances could be achieved by increasing the ability of primary care physicians at village clinics, particularly those early in their careers.

## AUTHOR CONTRIBUTIONS

**Xingyue Zhu**: Conceptualization; methodology; investigation; writing—original draft preparation; formal analysis. **Yang Chen**: Conceptualization; methodology; writing—review & editing. **Xingjiang Liao**: Conceptualization; investigation; writing—review & editing.

## CONFLICT OF INTEREST STATEMENT

The authors declare no conflict of interest.

## ETHICS STATEMENT

The study was conducted according to the guidelines of the Declaration of Helsinki, and approved by the Ethics Committee of Guizhou Medical University (protocol code: 2022‐292; date of approval: 13 Sep, 2022).

## INFORMED CONSENT

Informed consent was obtained from all subjects involved in the study.

## Supporting information

Supporting information.

## Data Availability

No data is available.
