# Supplementary material for: Factors underlying burnout among rural village physicians in Southwestern China
Source: Health Care Sci. 2023 Jul 26;2(4):233–41. doi: 10.1002/hcs2.62 (PMC11080832; doi:10.1002/hcs2.62)
Supplement: Supplementary file 1 — Supporting information. [file HCS2-2-233-s001.docx]

Table S1. List of the basic public health services

| Item | Population | Task and requirement |
| --- | --- | --- |
| 1. Resident health profile | All permanent residents within the district | 1. To file individual health profile for permanent residents within the district which includes basic personal information, physical examination, health management records of key populations and other medical and health service records. |
| 1. Health education | All permanent residents within the district | 1. To publicize information about healthy lifestyle, preventable risk factors as well as knowledge of major chronic noncommunicable diseases, and to improve health literacy 2. To issue printed material, and provide no less than 12 types of printed materials per year; 3. To establish health education bulletin, and change its content no less than evert two months; 4. To hold at least one talk about health education every two months. |
| 1. Prevention and vaccination | Children aged 0 to 6 years and other key populations | 1. To establish vaccination records; 2. To inform parents of vaccination affairs; 3. To vaccinate children. |
| 1. Health management for children aged 0 to 6 years | Children aged 0 to 6 years | 1. To visit neonates at one week after discharge and at one month; 2. To follow up infants aged below 3 years for 8 times; 3. To provide consultation, physical examination and developmental assessment every year for children aged 4-6 years. |
| 1. Maternal health management | Maternal residents | 1. To provide health assessment, physical examination along with guidance on lifestyle, mental health, exercise and nutrition. |
| 1. Health management for the elderly | Permanent residents aged 65 years and above | 1. To provide physical examination and assessments on lifestyle and health status; 2. To give specific recommendations or guidance. |
| 1. Non-communicable chronic diseases management | | |
| - 1. Hypertension health management   Population:  Task and requirement: | Hypertension patients aged 35 years and above | 1. To screen residents by providing blood pressure measurement ever year; 2. To follow up patients with primary hypertension face to face for at least 4 times per year; 3. To provide specific interventions, including medicine treatment adjustment, lifestyle prescriptions or referring to other facilities; 4. To conduct comprehensive physical examination for patients with primary hypertension for at least one time per year. |
| - 1. Type 2 diabetes mellitus health management | Type 2 diabetes patients aged 35 years and above | 1. To provide fasting blood glucose testing for populations with high risk of type 2 diabetes at least one time per year; 2. To provide fasting blood glucose testing for type 2 diabetes patients at least four times per year; 3. To provide specific interventions, including medicine treatment adjustment, lifestyle prescriptions or referring to other facilities; 4. To conduct comprehensive physical examination for type 2 diabetes patients for at least one time per year. |
| 1. Major psychiatric disorders health management | Patients with major psychiatric disorders who are clearly diagnosed and living at home among permanent residents in the district. Mainly including schizophrenia, schizoaffective disorder, paranoid psychosis, bipolar disorder, epilepsy-induced mental disorder, and mental retardation with psychotic disorder. | 1. To file patients; to follow up patients and conduct risk assessment, at least four times per year; 2. To provide specific interventions, including medicine treatment adjustment, guidance on rehabilitation or referring to other facilities; 3. To conduct comprehensive physical examination, at least one time per year. |
| 1. Tuberculosis health management | Patients with pulmonary tuberculosis | 1. To refer suspected patients to other facilities; 2. To visit patients when informed by the facilities issuing diagnosis certifications, and provide drug utilization information; 3. To supervise medication (by physicians or family members); 4. To follow up patients at least one time per month; 5. To provide specific interventions, including referring, and urge the subsequent visit to the facilities issuing diagnosis certifications; 6. To perform final assessment when the patient discontinued anti-tuberculous therapy. |
| 1. Traditional Chinese medicine health management | | |
| 10.1 TMC health management for the elderly | Permanent residents aged 65 years and above | 1. To provide one TMC health management service per year, including TMC physical identification and TMC healthcare guidance. |
| - 1. TMC health management children aged 0 to 6 years | Children aged 0 to 36 months | 1. To provide parents with guidance on TCM for children at 6, 12, 18, 24, 30, and 36 months of age, which includes: first, guidance on TCM diet and activities for children.; second, methods of abdominal massage and chiropractic at 6 and 12 months of age, and methods of acupressure at 18, 24, 30 and 36 months of age. |
| 1. Reporting of infectious diseases and public health emergencies | Residents within the jurisdiction | 1. To discover, register and report cases of infectious diseases and public health emergencies within stipulated timeline (two hours for Class A infectious diseases (and some Class B infectious diseases); twenty-four hours for most Class B infectious diseases); 2. To manage patients and suspected patients by means of separation and medical observation; 3. To assist epidemiology survey and track the close contacts; 4. To assist vaccination and preventive medication. |
| 1. Health supervision and contraceptive health management | Residents within the jurisdiction | 1. To report foodborne diseases; 2. To inspect drinking water; 3. To assist health supervision in schools and guide health education for students; 4. To report illegal medical practice; 5. To assist actions of family planning and contraception. |

Table S2. The results of the full linear regression on burnout score.

| Characteristic | Coefficient | *P* | 95% CI |  |
| --- | --- | --- | --- | --- |
| Sex |  |  |  |  |
| Male | 0[Reference] |  |  |  |
| Female | -1.53 | 0.241 | -4.20 | 1.14 |
| Education level |  |  |  |  |
| Technical school | 0[Reference] |  |  |  |
| Junior college | 0.22 | 0.776 | -1.40 | 1.84 |
| Bachelor degree | -0.01 | 0.996 | -2.99 | 2.98 |
| Title |  |  |  |  |
| No | 0[Reference] |  |  |  |
| Yes | 0.16 | 0.854 | -1.71 | 2.03 |
| Working years |  |  |  |  |
| $\leq$5 years | 0[Reference] |  |  |  |
| 6-10 years | -2.48 | **0.044** | -4.88 | -0.08 |
| 11-20 years | -2.65 | **0.034** | -5.07 | -0.22 |
| $>$20 years | -2.77 | **0.031** | -5.24 | -0.29 |
| Work hour per week |  |  |  |  |
| 40-49 hours | 0[Reference] |  |  |  |
| 50-74 hours | 1.72 | 0.289 | -1.62 | 5.07 |
| $\geq$75 hours | 1.36 | 0.524 | -3.08 | 5.79 |
| Workload for farm work |  |  |  |  |
| $<$one third of workload for village physician | 0[Reference] |  |  |  |
| $\geq$one third of workload for village physician | 4.60 | **0.040** | 0.25 | 8.96 |
| No farm work | -1.05 | 0.435 | -3.84 | 1.74 |
| Workload composition |  |  |  |  |
| Basic public health service dominated | 0[Reference] |  |  |  |
| Medical service dominated | -3.83 | **0.006** | -6.38 | -1.29 |
| Performance bonus |  |  |  |  |
| No | 0[Reference] |  |  |  |
| Yes | -0.18 | 0.884 | -2.83 | 2.47 |
| Unclear | 2.83 | 0.176 | -1.41 | 7.08 |
| No. of received trainings per year |  |  |  |  |
| $<$1 time | 0[Reference] |  |  |  |
| 1-3 times | -0.42 | 0.782 | -3.56 | 2.73 |
| $>$3 times | -0.08 | 0.953 | -2.91 | 2.75 |
| No. of received supports per year^*^ |  |  |  |  |
| $<$1 time | 0[Reference] |  |  |  |
| 1-5 times | -2.30 | **0.032** | -4.37 | -0.22 |
| $>$5 times | -5.01 | **0.029** | -9.45 | -0.58 |
| $R^{2}$ |  |  |  | 0.3841 |
| AIC | 454.1725 | | | |

^*^Supports included special consultations at village clinic by physicians from higher-level facilities, information share of testing and imaging within the alliance, and tele-medicine.
